# Electrical properties and chemiresistive response to 2,4,6 trinitrotoluene vapours of large area arrays of Ge nanowires

**DOI:** 10.1186/s11671-023-03780-1

**Published:** 2023-02-07

**Authors:** Paola Frigeri, Enos Gombia, Matteo Bosi, Giovanna Trevisi, Luca Seravalli, Claudio Ferrari

**Affiliations:** IMEM-CNR Institute, Parco Area delle Scienze 37/A, 43124 Parma, Italy

**Keywords:** Nanowire, Ge, NW–NW junctions, Electrical properties, Chemiresistive sensor, Explosive detection

## Abstract

We study the electrical and morphological properties of random arrays of Ge nanowires (NW) deposited on sapphire substrates. NW-based devices were fabricated with the aim of developing chemiresistive-type sensors for the detection of explosive vapours. We present the results obtained on pristine and annealed NWs and, focusing on the different phenomenology observed, we discuss the critical role played by NW–NW junctions on the electrical conduction and sensing performances. A mechanism is proposed to explain the high efficiency of the annealed arrays of NWs in detecting 2,4,6 trinitrotoluene vapours. This study shows the promising potential of Ge NW-based sensors in the field of civil security.

## Introduction

The development of sensors for the real-time vapour detection of explosives is of paramount importance to provide security controls for sensitive infrastructures. In addition to the most common detection methods currently employed in this field [1, 2], chemiresistive gas-phase sensors based on one-dimensional (1D) nanostructures have recently gained increasing interest mainly related to their intrinsically high surface-to-volume ratio and enhanced reaction activities [3]. 1D nanostructures including nanowires, nanotubes, and nanobelts can be potentially used to develop devices with unique sensing performances, low power consumption and easy integration with conventional circuits and portable devices. Compared with bulk materials, 1D materials have larger specific area and then more exposed active sites distributed on their surface. Due to high surface-to-volume ratio, the electrical properties of 1D nanostructures are significantly more sensitive to variations of the surface potential induced by adsorbed molecules on their surface. These features favour a fast charge transfer and then the development of nanosensors with high sensitivity and fast response capability [4].

Among 1D nanostructures, Si nanowires [5, 6] and metal oxide semiconductor nanowires [7] are the most explored for chemiresistive gas sensing applications. Nanowire (NW) sensors can be realized by assembling single NW, multiple NWs or networks of NWs and configured as simple resistors or as field effect transistors (FET).

According to our knowledge, there are relatively few studies on the electrical detection of explosive vapours based on 1D-nanostructures and these mainly concern the sensing properties of semiconducting metal oxide nanowires and Carbon nanotubes [8–12]. Good electrical sensing capability to explosive vapours has been also reported for devices based on Si NW, where the discriminative detection to different nitroaromatic species has been investigated by means of engineered arrays of coated [13] or molecular functionalised [14, 15] Si NWs configured as nano-FETs (Field Effect Transistors). Wang et al. [16] have shown that the plasma treatment of parallel Si NWs, fabricated by lithography process, can significantly improve the chemiresistive response to nitro explosives.

In all these studies regarding NW-based explosive detection, the nanoscale building blocks proposed to realize the vapour sensors are single nanowires. Nevertheless, also the assembly of random NWs deposited on suitable substrates, denoted as NW networks in this work, can be considered a system of interest for the development of innovative biological and light sensors exhibiting good performances such as high sensitivity and fast response and reset time [17–20]. The nanoscale structure of single NW components will ensure improved sensor properties, whereas the macroscopic features of NW networks make it possible the preparation of chip sensing devices with optimal stability and reproducibility by relatively simple and economic methods [21]. One of the main drawbacks of these systems is, however, related to the presence of native oxide on the surface of individual as-grown nanowires that hinders the electrical transport at NW/NW contacts causing a strong reduction in the device conductivity.

In this work we study the electrical properties and the chemiresistive response to 2,4,6 trinitrotoluene (TNT) vapours of large area Ge NW networks resulting from random disordered array of multiple nanowires deposited on insulating sapphire substrates by centrifuge method and subsequently subjected to an annealing treatment at the temperature of 450 °C in H_2_ atmosphere. The choice of the sapphire substrate is due mainly to its insulating property and to its excellent chemical and thermal stabilities. It is well recognized that Ge NWs [22] are an extremely interesting research topic due the unique properties of Ge at the nanoscale level as compared to Silicon, such as the higher carrier mobility and the enhanced carrier confinement, which are direct consequences of the small effective mass and of the large excitonic Bohr radius. Moreover, the processing of Ge can be done at a lower temperature as compared to Si, a feature which simplifies the integration of Ge nanostructures with conventional devices.

We demonstrate that Ge NW networks can be successfully employed for the development of explosive gas sensors with high sensitivity and fast response capability.

It is noteworthy to remark that although the surfaces of Ge NWs investigated in this work are not chemically modified, and despite the inherently low vapour pressure of few parts per billion of TNT at room temperature [23], sensors based on Ge NW networks have been shown to be extremely sensitive to TNT molecules.

## Methods/experimental

Ge nanowires were grown using an Au-catalysed chemical vapour deposition process with no intentional doping. Details of Metal Organic Vapour Phase Epitaxy (MOVPE) technique can be found in earlier works [24, 25]. Crystalline as-grown nanowires have diameters ranging from 70 to 90 nm and lengths up to 30 µm. Ge NW surfaces exhibit a thin (2–3 nm) native Ge oxide layer when exposed to air, as confirmed by transmission electron microscopy [24].

**Fig. 1:**
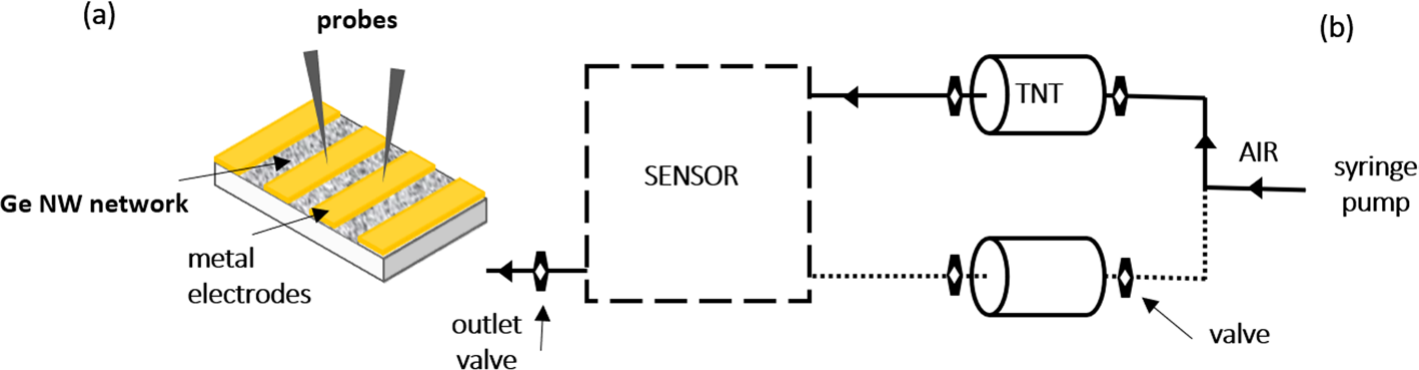
**a** Schematic of a Ge NW network deposited on sapphire substrate showing metal electrodes contacting the network; Au contact areas = 5 mm^2^, contact spacing = 1 mm. **b** Diagram of experimental setup used in sensing experiments

The deposition of Ge NW networks on sapphire substrates included the following steps: (i) sonification of as-grown Ge NWs for 30 s in ethanol, (ii) transfer of 5 cc of the solution into a tube containing the sapphire substrate at the bottom, and (iii) centrifugation at 6000 rpm for 10 min. The substrates with the NW networks were finally annealed at 450 °C for 30 min under H_2_ atmosphere (pressure of 60 mbar) with a flow rate of 500 sccm to remove the native oxide [26]. After the annealing process, Ge NW networks were quickly transferred into a vacuum chamber (5 × 10^–7^ Torr) for the thermal evaporation of the metal electrodes. The transfer process exposed Ge NW networks to ambient air for ∼ 20–30 min. Large area (1 × 5 mm) Au electrodes were realized with a 1 mm-sized inter electrode gap, as schematically shown in Fig. 1a. Au was used both for its ability to form ohmic contact on bulk p-type Ge and for its metallurgical stability [27]. During the same evaporation process, Au contacts were also deposited on non-annealed Ge NW networks used as reference structures to investigate the effects induced by the thermal treatment.

The morphological characterization of Ge NW networks was carried out by scanning electron microscopy (SEM) in a Zeiss Auriga Compact system equipped with a high-resolution Gemini column with a Schottky field-emission gun.

The electrical properties of NWs devices were investigated by two probes measurements at room temperature using a combination of a source/measurement unit (Keithley 2635B) and a probe station. To minimize the influence of electromagnetic fields as well as ambient light, the probe station is placed in the dark in a stainless-steel chamber.

The sensing response of Ge NW networks was monitored by measuring the resistance changes upon the exposure to TNT; the schematic diagram of the system used for sensing experiments is shown in Fig. 1b. A 10-ml plastic vial, loaded with 300 mg of solid TNT, acts as reservoir of TNT vapour. An automated syringe pump with a calibrated flow rate of 20 ml/min is used to transfer diluted TNT vapours from the reservoir into the test chamber. In addition, to validate the sensing results and to exclude the effect of unwanted environmental background signals, an alternative symmetrical line, indicated by the dotted line in Fig. 1b, was designed to transfer the air flow generated by the syringe pump to the sensor after passing through an empty vial of identical volume to that loaded with solid TNT.

## Results and discussion

### Morphological characterization of Ge NW networks

**Fig. 2:**
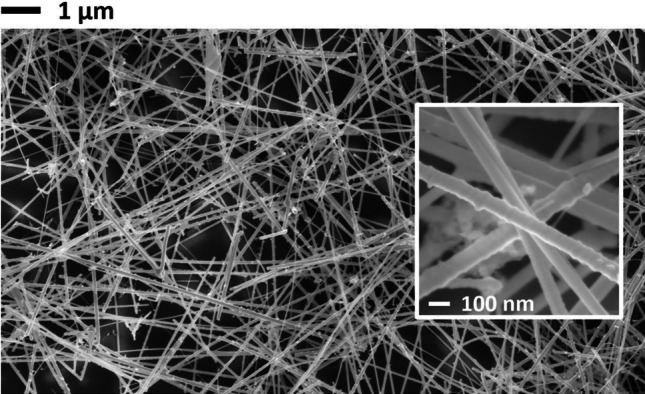
SEM image (scale bar = 1 µm) of an annealed Ge NW network; the inset shows a large-scale magnification (scale bar = 100 nm)

The morphological characterization of Ge NW networks was carried out performing SEM imaging (Fig. 2) on the areas in-between the Au electrodes. Annealed and non-annealed NW networks were analysed and compared with particular attention devoted to the characterization of NW/NW contacts. As a result of this study, we can affirm that, at least within the SEM resolution of 1.2 nm at 15 keV acceleration voltage, no visible morphology changes take place upon the annealing process. Therefore, the following information regarding the NW size applies to both samples. As expected from the study of the morphology of as-grown NW structures [24], the NW diameters measured in the network structures span approximately from 70 to 90 nm with a small fraction of NWs showing a reduced diameter in the 25–35 nm range. The major part of NWs shows a length that falls in the 10–15 µm range with only a few of them achieving more than 20 µm length. These values are lower as compared to the length of as-grown NWs. Surely the not-flat orientation of the NWs in the network structures with respect to the substrate induces a systematic error in the measured length, but it must be taken into account that also the sonication procedure used to detach the Ge NWs from the growth substrate may cause an effective reduction of the NW lengths.

### Electrical characterization of Ge NW networks

The electrical characterization of NW network devices was performed on the same set of samples used for SEM imaging. In agreement with previous studies on the photoresponse characteristics of devices based on Ge NWs networks [18–20], in all tested devices we observed a significant enhancement of the network conductivity when going from dark to illumination conditions. For this reason, all the measurements reported in this work were performed at least 2 h after the sample was placed inside the closed test chamber, a time that we have verified to be long enough to reach stable dark conditions.

**Fig. 3:**
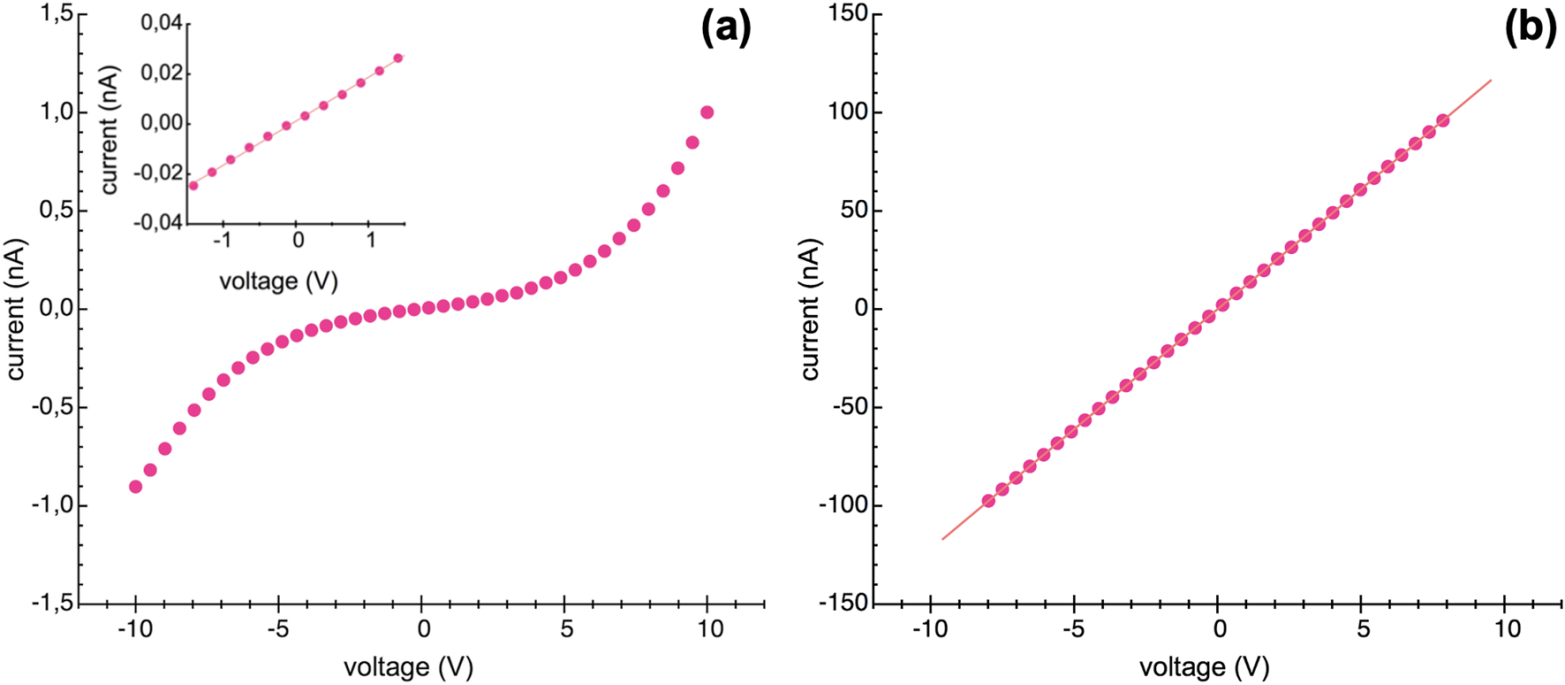
Dark *I*–*V* characteristics of the non-annealed **a** and annealed **b** Ge NW network devices, respectively. The inset in **a** shows the magnification of *I*–*V* curve at low bias (± 1.5 V). The line in **b** represents the linear fit of the data

Figure 3 shows typical current–voltage (*I*–*V*) characteristics obtained in dark conditions between two adjacent Au electrodes of non-annealed (Fig. 3a) and annealed (Fig. 3b) Ge NWs, respectively. The effect of the annealing process on the Ge NW network conductivity appears immediately evident. Non-annealed device shows an almost symmetric and nonlinear *I*–*V* characteristic. By contrast, a symmetric and linear *I*–*V* curve is obtained on the annealed device with current values much higher than those found in the non-annealed device in the same voltage range. It should be noted, in fact, that *I*–*V* curves in Fig. 3 are plotted with different limits of the current axes of 1.5 nA (Fig. 3a) and 150 nA (Fig. 3b), respectively.

**Fig. 4:**
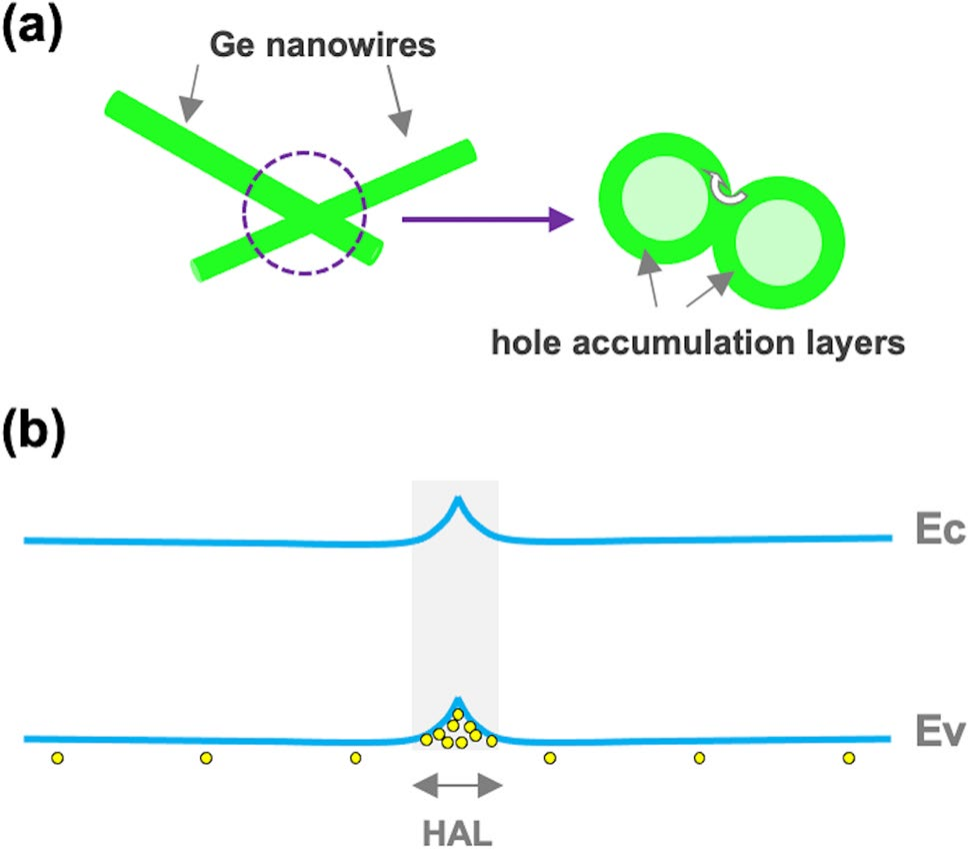
**a** Simplified illustration of two crossing Ge NWs and of the cross section at NW/NW contacts; the white arrow indicates the hole current path which occurs through the NW/NW interface and along the outer shells of Ge NW. **b** Schematic representation of energy band bending of NW/NW junction; Ev: top of valence band, Ec: bottom of conduction band, HAL: hole accumulation layer

To discuss in more detail these experimental findings, three main combined contributions to the electrical conductivity of the NW network have to be considered: (1) the conduction between Au electrodes and NW network, (2) the conduction along the NWs and (3) the conduction through NW/NW contacts. The first two contributions also affect the current transport in single NW-based devices [28], whereas the third characterizes the electrical properties of NW network devices. Moreover, it is noteworthy to mention that even though Ge NWs grown for this study are nominally undoped, it is well known that the presence of acceptor-like trap levels on Ge NW gives rise to the hole accumulation at the surface of NW [29–31]. In support to this hypothesis, in our previous investigations we observed that single Ge NW, obtained under the same growth conditions of NW structures studied in this work and contacted by focused ion beam (FIB)-deposited platinum [25], showed a p-type field effect behaviour. As a consequence, the semiconductor–metal contact formation at the electrodes and the conduction mechanisms through NW networks can be properly discussed assuming the p-type character for Ge NWs*.* At the interfaces between intersecting nanowires, the hole accumulation layers (HAL) are brought in contact (Fig. 4a) and the energy band bending at the NW/NW contacts can be schematically represented by the diagram in Fig. 4b, which has been obtained by neglecting the presence of insulating layers between contiguous NWs. Figure 4a also shows the current path of the holes which, after passing through the NW/NW junctions, flow predominantly along the outer shells of the Ge NWs.

In order to explain the electrical behaviour of non-annealed devices, however, the native oxide layer covering the surface of NW cannot be neglected. Such insulating layers lead to the formation of potential barriers both at metal electrodes/NW network interfaces and at the multiple NW/NW contacts. Non-annealed devices can be therefore described as two back-to-back connected Schottky barriers in series with the high resistive NW network*.* For low bias voltage (*V* < 1.5 V in Fig. 3a), the applied voltage drops mainly on the two Schottky barriers and the current trough the device is very low (~0.025 nA at 1.5 V). According to the general theory derived by Simmons [32] to describe the electric tunnel through a thin insulating layer, in this regime the contact resistances are almost independent from the applied voltage and the *I*–*V* curve exhibits a linear behaviour as shown in the inset of Fig. 3a. With increasing the bias voltage, the carrier tunnelling probability at the metal contacts increases, giving rise to the nonlinear behaviour of *I*–*V* curve. At moderately large bias (*V* > 8 V), as the potential barriers become thin enough for a significant tunnelling current at the metal/NWs network interfaces, the contact resistance at the electrodes strongly decreases and the applied voltage drops mainly on the NW network. In these conditions the transport at NW/NW junctions becomes the dominating mechanism and the *I*–*V* curve approaches a straight line, with a slope that allows for an estimation of the network resistance *R~*4.2 ÷ 5.1 GΩ, where 4.2 and 5.1 GΩ are the values calculated for positive and negative voltages, respectively. However, the current flowing through the device remains relatively low, mainly due to the presence of the highly resistive NW/oxide/NW junctions.

In contrast, the linear behaviour of *I*–*V* curve of annealed Ge NWs (Fig. 3b) proves that the annealing process, performed before the deposition of the Au contacts, clearly produces remarkable effects on the current conduction trough the NW network device. The thermal treatment, causing the effective thickness reduction of the oxide on the NW surface, gives rise to (i) an irreversible change in the electrical behaviour of the metal contacts from rectifying to ohmic and (ii) the formation of oxide-free channels for the current conduction at the NW/NW interfaces. The relatively low resistance *R* = 81.9 MΩ calculated from linear *I*–*V* characteristic in Fig. 3b, which is about two orders of magnitude lower than the values estimated from the linear regions of the *I*–*V* curve in Fig. 3a, seems to confirm the effectiveness of the annealing process in reducing the oxide layer thickness on Ge NWs.

These results demonstrate that the electrical properties of Ge NW networks show a strong dependence upon the annealing procedure, while, as reported above, no morphology changes were observed by SEM between annealed and non-annealed NW network. This suggests that the structural modifications induced by annealing must take place at the buried interface of the NW/NW contact, not accessible by SEM analysis, and/or concern the thin oxide layer that covers the NWs, whose presence is not detectable by SEM. Such observation was reported also by Ternon et al. [33] on Si NWs and was supported by high-resolution transmission electron microscopy (HRTEM) measurements, which reveal the formation of necks between contiguous NWs of diameter increasing with the annealing temperature. The same authors [21] reported that annealed Si NW networks show increased conductivity and that sintered NW/NW junctions are protected from oxidation also for prolonged exposure to air, ensuring high reproducibility and stability over months of the electrical characteristics of the networks.

In order to verify the long term stability of the annealed Ge network devices, we have monitored the time evolution of the electrical characteristics by comparing the initial *I*–*V* curve, acquired about 2 h (h) after the device fabrication with those obtained on the same device after ~48 and ~1000 h. A resistance increase of about 10% and 13.5% was measured after ~48 h and ~1000 h, respectively, indicating that the most significant modifications induced by the exposition to air occur within a relatively short period of time. We suggest that the oxide formation in the regions close to the necks connecting the NWs causes a reduction of the contact area of the multiple NW/NW junctions and therefore determines an increase in the resistance of the device up to a stable value, when the oxide reaches an almost steady thickness. These results demonstrate that the annealing process partially protects the contact regions between NWs from oxidation. This feature, as it will be discussed in the next section, represents a crucial aspect since the surface processes, induced by the interaction between TNT molecules and NW network, are expected to mainly affect the conduction through NW/NW contacts and only to a lesser extent the current transport along NWs in the network.

### Sensing experiments

To study the sensing properties of Ge NW network arrays, numerous test structures have been fabricated and experimentally tested. The effect of TNT vapours on device resistance has been measured by using the sensing setup and the procedure described in the METHODS/EXPERIMENTAL.

**Fig. 5:**
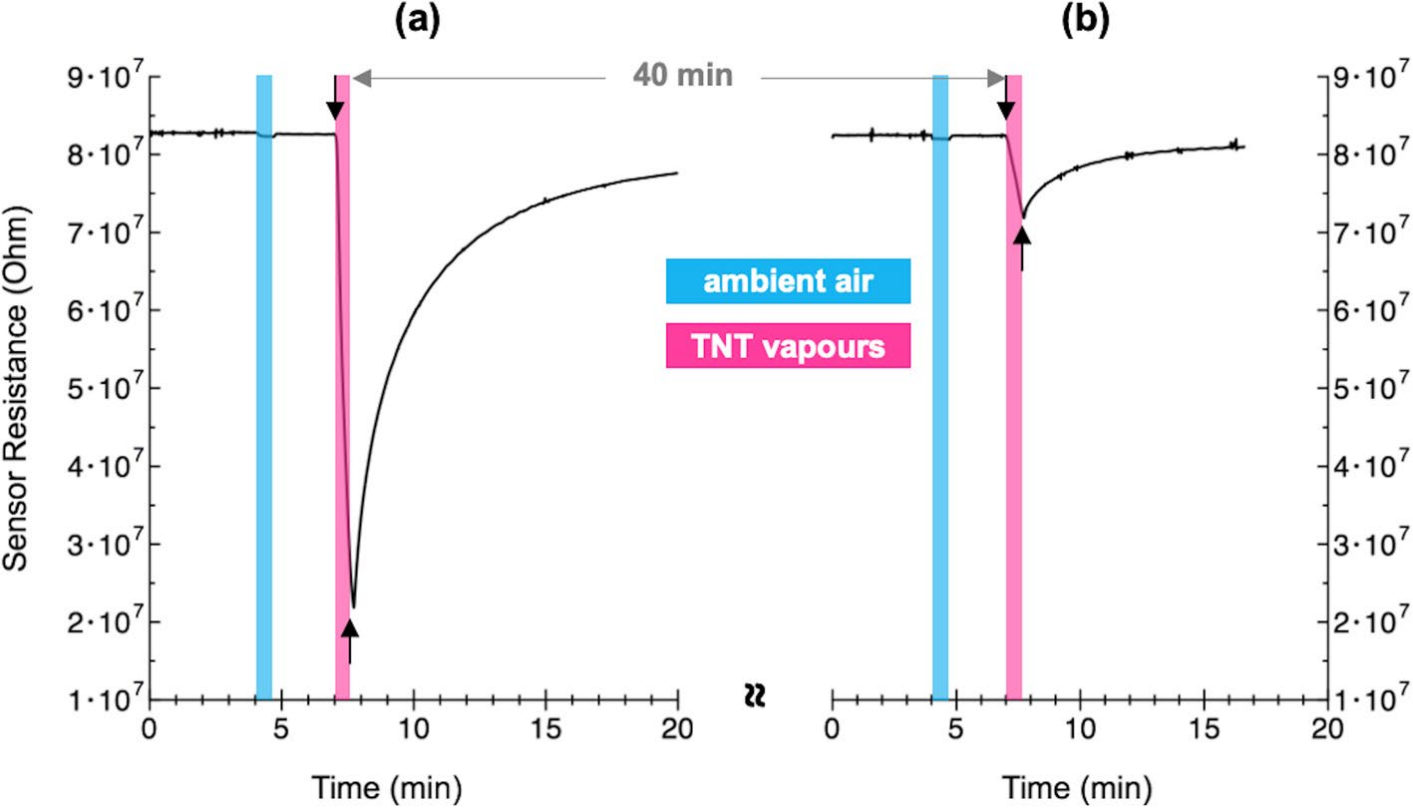
Typical time response of the Ge NW network to ambient air (light blue areas) and TNT vapours (pink areas) for 45 s. Downward and upward arrows indicate the start and stop of exposure time of NW network device to TNT vapours. The measurements were performed after saturated (**a**) and unsaturated (**b**) vapour concentration was reached inside TNT container

Figure 5 shows the typical time response at room temperature of an annealed Ge NW network device upon exposure to ambient air (light blue areas) and to TNT atmosphere (pink areas) for the same time of 45 s. The measurements in Fig. 5a were performed after the saturated vapour concentration was reached inside the TNT container (several hours). In this case, the TNT concentration in the mixed TNT-air vapour, *C*_TNT-air_ is ~1 ppb and can be roughly estimated by the equation:

$$C_{{{\text{ TNT}} - {\text{air}}}} \left( {{\text{ppb}}} \right) = C_{{{\text{TNT}}}} \left( {{\text{ppb}}} \right) \frac{{F _{{{\text{TNT}}}} }}{{F _{{{\text{TNT}}}} + F _{{{\text{air}}}} }}$$

where the saturated vapour concentration *C*_TNT_ of ~2.6 ppb has been assumed by extrapolation from Clausius-Clapeyron plot [23] at the laboratory temperature of 23.5 °C and *F*_TNT_ and *F*_TNT_ + *F*_air_ are the TNT and total flow rates, respectively.

As can be seen from Fig. 5a, the resistance change after exposing the sensor to air without TNT for 45 s is only ≈ 0.5%. Since the syringe pump used as the air source in the experiment was filled with ambient air, the small variation observed could be attributed to slight differences in temperature and humidity between the laboratory atmosphere, here defined as "air without TNT", and the atmosphere inside the test chamber. On the contrary, during TNT flow the sensor resistance is strongly reduced to ≈ 75% of its initial value, before gradually recovering after the mixed TNT-air stream is removed through a valve connected to an exhaust. The response time of the sensor, defined as the time taken to go from 10 to 90% of the total resistance change, is 27 s and the full baseline recovery after the TNT-air flow interruption, which takes about 1100–1200 s, proves the reversibility of adsorption/desorption process on the NW network surface.

In order to evaluate the sensor response to different TNT concentrations, after the one shown in Fig. 5a, a second run of measurements was carried out on the same device, before the saturated vapour concentration was reached inside the closed vial containing the TNT. Under these conditions we observe, as reported in Fig. 5b, an almost unchanged sensor response to ambient air flow (≈ 0.6%) with respect to the data in Fig. 5a, whereas the resistance change after exposing the sensor to TNT vapours is ≈ 13% of the baseline value, so testifying the lower TNT vapour concentration injected in the test chamber. It should be pointed out that the same large response to explosive vapours shown in Fig. 5a, was observed again by recovering the initial conditions of complete saturation of TNT vapours inside the closed vessel containing the solid TNT. Moreover, high sensitivity to TNT and similar time responses were also obtained for all the investigated devices after the thermal annealing process, while the untreated, non-annealed devices did not exhibit any measurable conductivity change imputable to the exposure to TNT vapours (not shown here).

A conceivable explanation of the high sensing response to TNT, can be found by assuming that the sensing mechanism in Ge NW network annealed devices is similar to the well-established working principle of metal oxide semiconductor chemiresistive gas sensors [34, 35]. We suggest that the absorption of TNT molecules on Ge NW network can lead to charge transfer from Ge NWs to electron-deficient nitroaromatic ring of TNT. A similar charge transfer mechanism was proposed to explain the sensing properties of TiO_2_(B) NWs [36] and Si NWs [16] exposed to vapours of nitro explosives under ambient conditions. In [36], the chemiresistive response of TiO_2_ NW is directly related to the presence of hydroxyl groups (OH) on the surface of TiO_2_ NWs. The interaction between OH groups and nitro groups in explosives would facilitate the surface physisorption and the charge transfer processes. This evidence is confirmed by Fourier transform infrared spectroscopy (FTIR) investigations showing the formation of a complex between nitro groups bonded to TiO_2_ through hydroxyl groups. The role of OH groups in the charge transfer process is moreover supported by the experimental evidence that a higher density of hydroxyl groups on the surface of TiO_2_ leads to an increased response of the sensor.

We suggest that the contribution of OH groups could also explain the interaction between TNT molecules and Ge NW networks. To support this hypothesis, it is should be pointed out that after the annealing process, the long exposure of Ge NW networks to air during the test device fabrication and the measurements runs, is expected to cause the new oxidation of the Ge NW surface. On the contrary, the results of the electrical characterization reported above seem to demonstrate that the channels at the NW/NW interfaces remain oxide-free. Wang et al. [26] studied the oxide formation on Ge NWs by X-ray Photoelectron Spectroscopy (XPS) measurements and shown that, upon the exposure to air of clean p-type Ge NW, an oxide layer consisting mainly of GeO is formed in few minutes. Longer air exposure (hours) causes the formation of a GeO_2_ layer that strongly adsorbs H_2_O molecules. The authors also concluded that the hysteresis in gate response of Ge NW-based FET is mainly due to the water molecules strongly bound to GeO_2_ surface. Theoretical studies [37], developed to evaluate the causes of the electrical hysteresis in Ge NW-based devices, confirmed this conclusion showing that the absorption of water molecules on the Ge NW surface would lead to the formation of Ge NWs with terminal OH groups. As reported for TiO_2_ [36] and SI NW [16], the hydroxyl groups on the surface of Ge NWs could have a main role in the transfer process between Ge NWs and TNT molecules responsible of the chemiresistive effect.

**Fig. 6:**
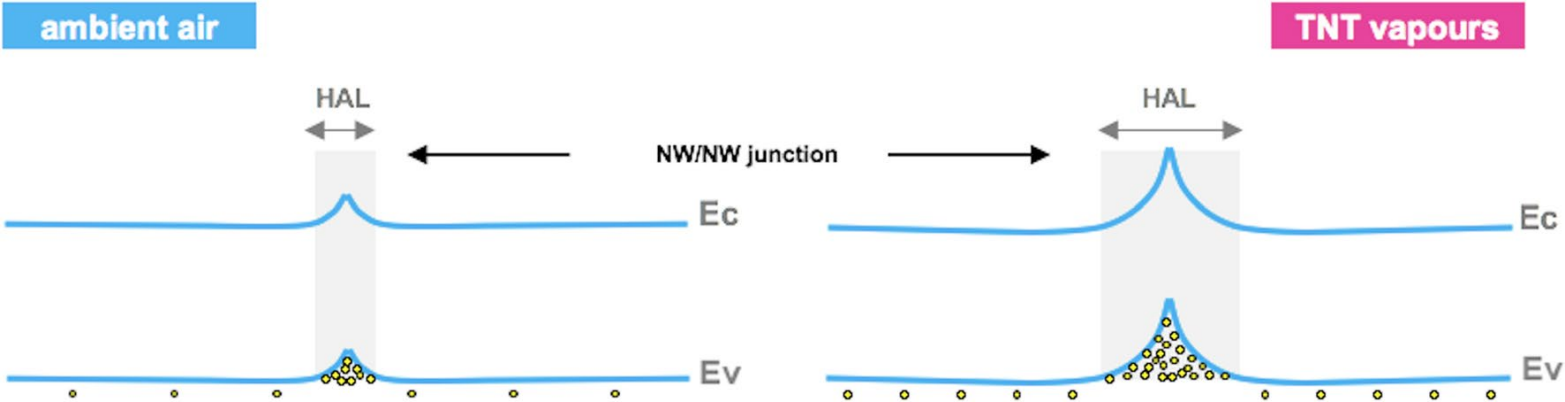
Energy band bending at NW/NW junctions and schematic diagram of gas sensing mechanism. Ev: top of valence band, Ec: bottom of conduction band, HAL: hole accumulation layer

By considering as previously discussed, the p-type character of Ge NWs, the interaction between TNT and Ge NWs causes an increase of the hole accumulation widths and then induces a larger upward bending of the energy bands near the surface of the nanowires (see Fig. 6), with the main effect to decrease the resistance of the contacts between NWs, since the resistance changes induced by the modulation of hole accumulation width along the NWs are expected to have minor effects on the total network resistance [38]. As a consequence, the regions which are mainly affected by surface processes are the regions close to numerous NW/NW contacts which act as active sensing sites and give rise to the observed high sensitivity to TNT vapours.

## Conclusions

In summary we prepared Ge NW networks on sapphire substrates and annealed them at 450 °C in H_2_ atmosphere. Planar Au electrodes were then deposited on the NW networks in order to obtain simple devices which were used to investigate the effect of the annealing on the electrical characteristics of the networks and to test their sensing properties to TNT vapour.

We showed that the annealing induces a significant modification of the *I*–*V* characteristics of the Au/NW network/Au structures and a consistent increase of the conductivity with respect to non-annealed networks, which has been mainly attributed to the surface oxide reduction at the NW/NW contacts. Moreover, the devices prepared on annealed NW networks exhibited a strong reduction of the resistivity of about 75% when exposed to TNT vapour in contrast to the devices obtained on non-annealed networks which did not show any detectable variation in resistance.

We concluded that electrical and sensing properties of Ge NW devices are mainly controlled by the features of multiple NW junctions in the network. A sensing mechanism similar to that reported for metal oxide semiconductor-based sensors is proposed to explain the high sensitivity and fast response observed in Ge NW networks which have undergone the annealing process.

The explosive detection system proposed in this work offers several advantages, such as the easy device fabrication and the relatively simple configuration of the experimental setup used to measure real-time resistance changes upon exposure to TNT vapours. The main drawback is related to the lack of a quantitative analysis of the TNT vapours. The promising results obtained stimulate future research aimed at optimizing the preparation of the Ge NW devices, improving the measurement methodology and testing the sensor with different vapour species, in order to verify its selectivity.

## Data Availability

The datasets used and/or analysed during the current study are available from the corresponding author on reasonable request.

## Abbreviations

NW: Nanowire
1D: One-dimensional
FET: Field effect transistor
TNT: Trinitrotoluene
MOVPE: Metal organic vapour phase epitaxy
SEM: Scanning electron microscopy
*I*–*V*: Current–voltage
FIB: Focused ion beam
HAL: Hole accumulation layer
HRTEM: High-resolution transmission electron microscopy
FTIR: Fourier transform infrared spectroscopy
XPS: X-ray photoelectron spectroscopy
